# The potential role of cost-utility analysis in the decision to implement major system change in acute stroke services in metropolitan areas in England

**DOI:** 10.1186/s12961-018-0301-5

**Published:** 2018-03-14

**Authors:** Rachael M. Hunter, Naomi J. Fulop, Ruth J. Boaden, Christopher McKevitt, Catherine Perry, Angus I. G. Ramsay, Anthony G. Rudd, Simon J. Turner, Pippa J. Tyrrell, Charles D. A. Wolfe, Stephen Morris

**Affiliations:** 1 Research Department of Primary Care and Population Health, Royal Free Medical School, University College London, Royal Free Campus, Rowland Hill Street, London, NW3 2PF United Kingdom; 2 Department of Applied Health Research, University College London, 1-19 Torrington Place, London, WC1E 7HB United Kingdom; 3 Alliance Manchester Business School, University of Manchester, Booth Street West, Manchester, M15 6PB United Kingdom; 4 Division of Health & Social Care Research, School of Medicine, King’s College London, Capital House 7th Floor, 42 Weston Street, London, SE1 3QD United Kingdom; 5 Guy’s and St Thomas’ NHS Foundation Trust, St Thomas’ Hospital, London, SE1 7EH United Kingdom; 6 Centre for Primary Care, Division of Population Health, Health Services Research and Primary Care, School of Health Sciences, Faculty of Biology, Medicine and Health, University of Manchester, Oxford Road, Manchester, M13 9PL United Kingdom

**Keywords:** economic evaluation, stroke, major system change, implementation, cost-effectiveness, programme budgeting and marginal analysis

## Abstract

**Background:**

The economic implications of major system change are an important component of the decision to implement health service reconfigurations. Little is known about how best to report the results of economic evaluations of major system change to inform decision-makers. Reconfiguration of acute stroke care in two metropolitan areas in England, namely London and Greater Manchester (GM), was used to analyse the economic implications of two different implementation strategies for major system change.

**Methods:**

A decision analytic model was used to calculate difference-in-differences in costs and outcomes before and after the implementation of two major system change strategies in stroke care in London and GM. Values in the model were based on patient level data from Hospital Episode Statistics, linked mortality data from the Office of National Statistics and data from two national stroke audits. Results were presented as net monetary benefit (NMB) and using Programme Budgeting and Marginal Analysis (PBMA) to assess the costs and benefits of a hypothetical typical region in England with approximately 4000 strokes a year.

**Results:**

In London, after 90 days, there were nine fewer deaths per 1000 patients compared to the rest of England (95% CI –24 to 6) at an additional cost of £770,027 per 1000 stroke patients admitted. There were two additional deaths (95% CI –19 to 23) in GM, with a total costs saving of £156,118 per 1000 patients compared to the rest of England. At a £30,000 willingness to pay the NMB was higher in London and GM than the rest of England over the same time period. The results of the PBMA suggest that a GM style reconfiguration could result in a total greater health benefit to a region. Implementation costs were £136 per patient in London and £75 in GM.

**Conclusions:**

The implementation of major system change in acute stroke care may result in a net health benefit to a region, even one functioning within a fixed budget. The choice of what model of stroke reconfiguration to implement may depend on the relative importance of clinical versus cost outcomes.

**Electronic supplementary material:**

The online version of this article (10.1186/s12961-018-0301-5) contains supplementary material, which is available to authorized users.

## Background

Major system change involves the reorganisation, sometimes called reconfiguration, of services at a regional level. Hospital centralisation is a form of major system change where services are reorganised so that a reduced number of hospitals provide specialist clinical care. A common service reconfiguration is a hub and spoke model, where healthcare services provided in specialised hubs are complemented by a number of smaller services providing less specialist, complex or intensive care.

In 2010, a major system change of acute stroke services was implemented in two metropolitan areas in the English National Health Service (NHS), London (population 8.17 million) [1] and Greater Manchester (GM) (population 2.68 million) [1] with the aim of improving access to high quality specialist stroke care and thereby improve patient outcomes. In London, post reconfiguration, all patients identified as potentially having a stroke were taken by ambulance to one of eight hospitals with a designated hyper-acute stroke unit (HASU), a ward that provides intensive, dedicated stroke care from a specialist multi-disciplined team. Patients remained on the HASU for 72 hours before either being discharged or moved to one of 24 stroke units [2–6] that provide ongoing care and rehabilitation. In GM, a similar model was implemented with the exception that patients with suspected strokes were transferred to a hyper-acute centre only if the onset time was within the previous 4 hours.

The centralisations have been associated with a 1.1% reduction of stroke-related deaths at 90 days in London, or 168 fewer deaths 21 months after the centralisation, as well as a reduction in bed days of 1.4 days per patient in London and 2.8 bed days per patient in Manchester compared to the rest of England [4].

The centralisation of acute stroke care in GM and London provides an opportunity to explore the implications for decision-makers of two different ways of reporting the results of an economic evaluation of major system change, in particular contrasting ways of reporting quality adjusted life years (QALYs) versus costs.

### Economic evaluations and the decision to implement major system change

Economic evaluations of the implementation of major system change and policy initiatives are rare, with no specific framework-guiding analysis methods [7].

The decision to implement new interventions in publicly financed healthcare systems that operate within a fixed budget, as is the case with the NHS, can sometimes result in a need to weigh up clinical effectiveness and cost. An intervention shown to be clinically effective may cost significantly more than current practice. If the new, more expensive intervention is to be implemented, a decision may need to be made if (1) the healthcare service will now treat fewer patients as the cost per patient has now increased, or (2) funds need to be taken from another service to finance the cost of the new treatment. Alternatively, the new service and its clinical effectiveness may result in cost savings produced elsewhere and hence the total cost per patient is reduced.

To help payers and policy-makers decide whether they should implement a new technology or service there are a number of types of economic analysis that can be undertaken. Within the English NHS the most common type of economic analysis for evaluating if a new technology should be implemented is to report the incremental cost per QALY gained of current or best practice compared to a new technology. QALYs are the preferred unit of reporting effectiveness of the National Institute for Health and Care Excellence (NICE), the NHS government body responsible for evaluating new technologies and recommending their implementation, given that QALYs are a combination of both mortality and morbidity over time weighted for the general public preferences for health outcomes [8]. If the incremental cost to the NHS of a QALY gained as a result of the new technology is less than £20,000 per QALY, in 87% of cases, NICE will recommend that the new technology is made available to patients in the NHS [9]. However, the use of the £20,000 threshold has been criticised given that the cost to produce an additional QALY in the NHS is £12,936. As a result, approval of new technologies using a threshold higher than £12,936 risks displacing technologies where the cost to produce an additional QALY is less and hence more efficient [9].

An alternative approach for evaluating the impact of implementing new technologies or services is Programme Budgeting and Marginal Analysis (PBMA). PBMA compares the additional benefit achieved per pound spent on a technology or service, A, compared to another service or technology, B, where B may be current practice or even an alternative healthcare service. The benefit of PBMA is that it facilitates the evaluation of benefit of A compared to B across programmes of work and within a fixed budget [10].

The aim of this paper is to report the economic implications of the reconfiguration in London and GM compared to metropolitan areas in the rest of England. The information will be reported in a way to aid with the decision to implement different reconfigurations of acute stroke care using different types of analysis (1) reporting the incremental cost per QALY gained as recommended by NICE and (2) using a hypothetical region with a fixed budget and PBMA.

## Methods

### Overview

We estimated difference-in-differences in costs, including the cost of implementation, and clinical outcomes to evaluate the effect of the reconfigurations in London and GM on costs, mortality and QALYs compared to metropolitan areas in the rest of England. Metropolitan areas were chosen so that population density and blue light ambulance travel time to hospital were as comparable as possible with London and GM. The analysis was undertaken with a cost perspective of the English NHS and personal social services. Eight decision analytic models were developed, each with 1000 hypothetical stroke patients. The time periods were as in Morris et al. [4] as described below.

1.  London before the reconfiguration (January 2008 to January 2010)
2.  London after the reconfiguration (July 2010 to March 2012)
3.  The rest of England (Metropolitan only), excluding GM for the same time period as (1)
4.  The rest of England (Metropolitan only), excluding GM for the same time period as (2)
5.  GM before the reconfiguration (January 2008 to November 2008)
6.  GM after the reconfiguration (April 2010 to March 2012)
7.  The rest of England (Metropolitan only), excluding London for the same time period as (5)
8.  The rest of England (Metropolitan only), excluding London for the same time period as (6)

Total costs and QALYs per 1000 patients were calculated for each of the eight models at 90 days and 10 years after admission, using a discount rate of 3.5% for costs and QALYs [8]. Costs are for the year 2013/2014 and are in British Pounds (£). We report the difference in costs and QALYs for (1) and (2) compared to (3) and (4), and the difference in costs and QALYs for (5) and (6) compared to (7) and (8).

### Data

We used routinely collected hospital data (Hospital Episode Statistics (HES)), audit data (National Sentinel Stroke Clinical Audit 2008, Stroke Improvement National Audit Programme (SINAP)) and data from the published literature to construct the model. Table 1 provides details on the number of stroke patients in each dataset.

**Table 1:** Data sources and numbers

	GM		England comparison		London		England comparison	
	Before	After	Before	After	Before	After	Before	After
HES	3503	7685	42,880	95,244	15,276	15,023	100,511	84,801
Age, mean	74.3	73.9	75.8	75.3	73	73.3	75.7	75.3
> 75, %	56%	53.6%	61.2%	59.18%	54.3%	54.4%	60.7%	52.3%
Female, %	52.6%	50.4%	53.0%	53.2%	51%	49.8%	53.0%	52.2%
White British ethnic group, %	82.9%	84.2%	82.4%	86.5%	58.5%	55%	83.9%	86.5%
Intracerebral haemorrhage, %	11.5%	11.7%	13.0%	12.7%	15.7%	14.8%	12.9%	12.7%
Cerebral infarction, %	61.6%	64.4%	62.7%	71.3%	68.9%	76.1%	64.4%	71.2%
Stroke not specified, %	26.9%	23.9%	24.3%	16.1%	15.4%	9.1%	22.7%	15.8%
Charlson index, mean score	2.0	2.0	1.9	1.9	2.0	2.0	1.9	1.9
Most deprived fifth, %	8.4%	10.3%	17.1%	17.6%	12.6%	13.2%	17.3%	17.6%
SINAP		10,295		9044		16,553		9044
Age, mean		73.2		73.6		72.7		73.6
> 75, %		50%		51%		50%		51%
Female, %		51%		51%		49%		51%
Intracerebral haemorrhage, %		11%		11%		11%		11%
Cerebral infarction, %		89%		89%		89%		89%
Sentinel	653		537		1541		537	
Age, mean	74.5		74.6		73.3		74.6	
> 75, %	55%		53%		51%		53%	
Female, %	52%		52%		50%		52%	
Intracerebral haemorrhage, %	13%		11%		14%		11%	
Cerebral infarction, %	87%		89%		86%		89%	

*GM* Greater Manchester, *HES* Hospital Episode Statistics, *SINAP* Stroke Improvement National Audit Programme

The HES data only contained patients with a primary diagnosis of stroke, as defined by ICD-10 codes I61 (intracerebral haemorrhage), I63 (cerebral infarction) and I64 (stroke, not specified as haemorrhage or infarction), and from metropolitan areas using the urban/rural classification for England [11]. It is linked to data from the Office of National Statistics vital statistics to obtain date of death [12].

Sentinel was a national biennial audit that collected data on the first 60 strokes across participating hospitals for the period April to August each year. Data from the 2008 Sentinel audit were used [13]. SINAP was a national audit of stroke care across participating acute hospitals in England providing data from all identified patients in participating hospitals from July 2010 to 2012 [14].

The South London Stroke Register (SLSR), a population-based stroke prospective registry recording all first-ever strokes in patients of all ages living in an area of South London [15], was used to calculate changes in disability before and after the reconfigurations.

Further details on the datasets used can be found in Morris et al. [4] for HES data, Ramsay et al. [5] for Sentinel and SINAP, and Hunter et al. [3] for SLSR. Data were analysed using STATA 13.

### Model structure

**Fig. 1:**
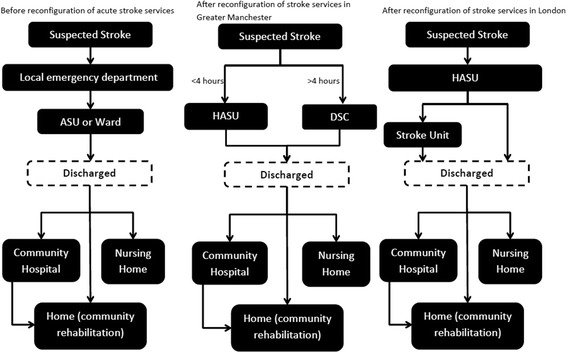
Stroke pathway in London and Greater Manchester: before and after reconfigurations. ASU acute stroke unit, HASU hyper acute stroke unit, DSC district stroke centre

Each of the eight models has two components:

1. A 90-day discrete event simulation of daily acute hospital ward movements, discharge destinations and mortality using data from HES, Sentinel and SINAP (Fig. 1).
2. A 10-year Markov model [16] with 90-day cycles using information from the 90-day model plus HES, the SLSR and published data to calculate costs and QALYs (Additional file 1: Figure S1).

All patients enter the 90-day model as an admission to hospital following a stroke. The proportion of patients in each health state in the first cycle of the 10-year model is determined by the final destination of patients in the 90-day model, including still on an acute hospital ward. The 90-day discrete event simulation is also used for 90-day costs and outcomes of hospitalisation following recurrent strokes in the 10-year Markov model.

**Fig. 2:**
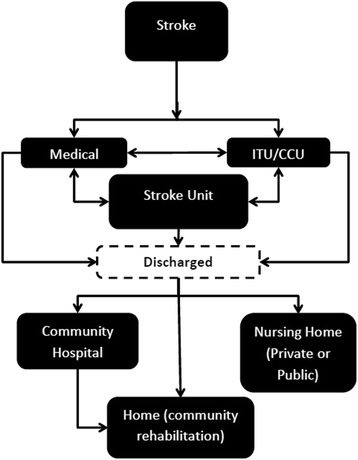
The 90-day discrete event simulation model structure. *ITU* intensive therapy unit, *CCU* coronary care unit

The flow for the 90-day model is illustrated in Fig. 2, excluding the state of death – patients have a probability of death every day of the 90-day model regardless of where they are in the model. The same flow is used for all eight models and is different to the stylised diagrams in Fig. 1. This is because, following the reconfigurations, despite the fact that patients should only have been admitted to a HASU in London and to a HASU if the stroke had occurred within 4 hours prior to admission and a District Stroke Centre if it had occurred more than 4 hours prior in GM, data from SINAP show only whether the first ward of admission was a stroke unit or not and do not provide information on the specific type of stroke unit [5].

The model was developed in Microsoft Excel 2010.

### Mortality

Mortality at 72 hours, 30 days and 90 days was calculated using the same data and a similar method to Morris et al. [2], including coefficients to adjust for differences between regions in age, sex, ethnicity, deprivation (index of multiple deprivation quintile) [17], and co-morbidities using the Charlson index [18]. Random effects for provider were also applied to the model. Two logistic regression models were fitted to the HES data; one for London and the rest of England excluding GM and one for GM and the rest of England excluding London. Values for the models are reported in Additional file 1: Table S1 and further details for the calculation of transition probabilities are provided in Additional file 1: Methods S1.

### Length of stay (LOS) and discharge destination

HES data were used to calculate the daily probability of discharge from acute hospital and discharge destination using a parametric survival model and Weibull distribution [16]. Two analyses, London (analyses 1 to 4 in overview) and GM (analyses 5 to 8 in overview), were run with the same adjustments as the mortality models (Additional file 1: Table S3). We assumed the probability of discharge was the same regardless of ward type as there is no information in HES on ward-specific LOS.

The method for calculating ward movements and discharge destination is provided in Additional file 1: Methods S2 and Tables S4 and S5.

### 10-year model

The model is described in Additional file 1: Figure S1 and model inputs in Additional file 1: Table S4. The possible states in the 10-year model are (1) home and in one of five health states based on the Barthel index, a measure of functional independence, where 0 is no functioning and 20 is fully functional (0–4, 5–9, 10–14, 15–19 and 20); (2) residential care or nursing home; (3) recurrent stroke and in hospital < 90 days; (4) recurrent stroke and in hospital > 90 days; and (4) dead.

The probability of patients discharged to community hospital or home being admitted to residential care or nursing home was taken from SLSR (Additional file 1: Table S4).

The probability that patients had a recurrent stroke following discharge was calculated using HES data and parametric survival analysis. A Weibull model [16] was used to give differential probabilities over time with probabilities calculated for 90-day cycles. Models were adjusted using the same methodology as above and were calculated for GM versus the rest of urban England, excluding London, and London compared to the rest of urban England excluding GM (Additional file 1: Table S4).

The probability of recurrent stroke for patients discharged in residential care or a nursing home was calculated from HES for patients discharged to residential care only.

The 10-year model takes into account the health and well-being of patients discharged from acute care using the Barthel index, with patients divided into the five Barthel states given above. The proportion of patients in each Barthel state and movement between them has been taken from the SLSR with different rates before and after (Additional file 1: Table S4). No data are available for Barthel indexes or any other measure of functioning or quality of life for GM or the rest of England before the reconfiguration. As a result, it is not possible to evaluate the impact of the reconfigurations on functioning or quality of life in GM or compare the changes that occurred in London with what happened over the same period in the rest of England. For the purpose of the model and calculating QALYs, the conservative assumption was made that the same improvements were seen in GM, the rest of England and London as were seen in the SLSR.

Probability of dying while in a care home was updated to Gordon et al. [19] as the most recent estimate available in the literature.

### Unit costs

Details of unit costs are given in Table 2. The cost of transfer has not been included as transfer costs were included in the additional cost of the HASU [6].

**Table 2:** Cost inputs for 90-day and 10-year model

Cost input	Cost per event/per day	Reference
HASU London uplift tariff day 1	£665^a^	London Stroke Strategy [6]
HASU London uplift tariff days 2–3	£399^a^	London Stroke Strategy [6]
GM Best Practice tariff per day for first 72 hours if admitted to hyper-acute care	£580	2014/2015 DH National Payment system [30] GM Guidance [31]
Stroke unit per bed day cost	£238	2013/2014 Reference Costs [32]
Medical assessment ward	£187	2013/2014 Reference Costs [32]
General medical ward	£218	2013/2014 Reference Costs [32]
Intensive care or critical care unit	£1578	2013/2014 Reference Costs [32]
Other ward not otherwise specified	£203	2013/2014 Reference Costs [32]
Nursing home	£105	PSSRU 2014 [33]
Private nursing home	£107	PSSRU 2014 [33]
Transfer to other NHS hospital (not acute)	£100	PSSRU 2014 [33]
Thrombolysis	£828	2014/2015 DH National Payment system [30]
90-day costs Barthel score 20–10	£459	Franklin et al. 2014 [34]
90-day costs Barthel score 0–9	£1926	Franklin et al. 2014 [34]
90-day costs residential care or nursing home	£10,647	Gordon et al. 2014 [19]

^a^Converted to 2013/2014 prices using Hospital and Community Health Services inflation index [33]

*DH* Department of Health, *GM* Greater Manchester, *HASU* hyper-acute stroke unit

### Costs of implementation

We undertook reviews of documentary evidence to identify the costs of implementing the reconfigurations in London and GM, including one-off financial investments to improve services, research to investigate the optimal configuration of services, and public and staff consultations.

### QALYs

QALYs were calculated using Barthel index from the SLSR and the methods described in Hunter et al. [3]. Utility scores were applied to each ward and discharge location and calculated as a daily rate for the 90-day model and each location for the 10-year model and calculated as a 90-day rate. Details on utility scores are provided in Additional file 1: Table S5.

### Cost-utility analysis

A difference-in-differences approach was used to compare cost and mortality at 90 days and costs and QALYs at 10 years; we compared costs, mortality and QALYs in London before and after the reconfiguration outcomes and costs over the same period in the rest of England (excluding GM). The same analysis was then conducted for GM (excluding London). Mortality and LOS was adjusted for differences between London, GM and the rest of England in age, sex, co-morbidities (Charlson Index [18]), stroke type and level of deprivation [17].

Net monetary benefit (NMB) is defined as total QALYs multiplied by a willingness to pay (WTP) for a QALY minus costs. Incremental NMB is calculated for London before the changes compared to after (models 1 and 2) and England, excluding GM, for the same time periods (models 3 and 4) for a range of values of WTP for a QALY gained (£0 to £100,000). If the incremental NMB was higher for London than the rest of England the London reconfiguration was then considered to be cost-effective. The same was performed for GM.

### PBMA

The budget for delivering acute stroke care in the hypothetical example was set at £40 million pounds per year, equating to the approximate amount required to treat 4000 strokes a year, close to the average number of admissions to acute hospital with a diagnosis per year in a region of approximately 3 million inhabitants in England [20]. Using the goal seek command in Excel, we calculated the number of patients with stroke treated in 1 year and total QALYs at 1, 5 and 10 years if a region has a fixed budget of £40 million per year. This was calculated before and after reconfiguration and differences between reconfigured areas and the rest of metropolitan England were compared. The cost per patient of implementation was included in the analysis.

### Probabilistic sensitivity analysis

Probabilities were applied to the model using the methodology given in Briggs et al. [16]. For costs, a gamma distribution was used with 25% variation around the mean. A total of 5000 iterations of the eight models were run and costs, mortality and QALYs over 10 years and 90 days were captured. The percentage of iterations where London or GM had a higher NMB than the rest of England for a specific WTP for a QALY gained were recorded and shown on a cost-effectiveness acceptability curve. Additionally, 95% confidence intervals were calculated using the standard deviation of the 5000 iterations of the model.

### Other sensitivity analyses

To test assumptions made in the model, five sensitivity analyses were conducted. Details of the sensitivity analyses conducted are reported in Additional file 1: Methods S1.

## Results

### Base case

At 90 days, London had a reduced risk of mortality compared to the rest of England before versus after the reconfiguration. Per 1000 strokes, the adjusted number of deaths in London at 90 days was 100 before the reconfiguration and 75 after, thus a reduction of 2.7 percentage points in the number of deaths. During the same period in England there was an adjusted reduction in deaths of 1.8 percentage points (114 deaths before compared to 97 deaths after) (Table 3). This represents a relative reduction in deaths in London compared to the rest of England of 0.9% or 9 deaths per 1000 patients. In 90% of iterations of the model, London had a greater reduction in deaths after the reconfigurations than the rest of England. Compared to the rest of England, there was no reduction in deaths in GM at 90 days. Both areas had a reduction in LOS relative to the rest of England, namely of 2 days less in GM and 0.6 days less in London.

**Table 3:** Mean and 95% confidence intervals (CIs) for costs, mortality and QALYs, at 90 days and 10 years for London per 1000 patients

	London			England			DID
	Before	After	Difference	Before	After	Difference	
90-day results							
Deaths	100 (80 to 120)	75 (56 to 92)	−25 (−41 to − 10)	114 (93 to 133)	97 (79 to 115)	−17 (−21 to − 12)	−9 (−24 to 6)
LOS	19 (18 to 20)	16 (15 to 17)	−3 (−4 to −3)	20 (18 to 21)	17 (16 to 18)	−3 (−3 to −2)	−1 (−1 to 0)
QALYs	102 (96 to 108)	110 (88 to 132)	8 (−14 to 29)	100 (93 to 106)	104 (98 to 110)	4 (4 to 5)	3.6 (−18 to 25)
Costs	£5,705,774 (£4,598,498 to £6,813,049)	£5,949,155 (£5,069,827 to £6,828,484)	£243,381 (−£182,658 to £669,421)	£5,492,431 (£4,394,421 to £6,590,440)	£4,965,785 (£3,956,766 to £5,974,803)	−£526,646 (−£640,210 to −£413,081)	£770,027 (£392,152 to £1,147,902)
10-year results							
Deaths	386 (316 to 455)	360 (289 to 432)	−25 (−38 to −13)	417 (351 to 483)	401 (329 to 475)	−15 (−44 to 13)	−10 (−37 to 17)
QALYs	2931 (2499 to 3363)	3473 (3079 to 3867)	542 (109 to 975)	2802 (2391 to 3214)	3287 (2909 to 3665)	484 (66 to 903)	58 (−76 to 193)
Costs	£39,459,874 (£29,343,049 to £49,576,698)	£38,146,542 (£27,941,281 to £48,351,802)	–£1,313,332 (−£5,315,567 £2,688,902)	£39,818,504 (£29,562,087 to £50,074,921)	£37,490,809 (£27,170,660 to £47,810,958)	–£2,327,696 (−£6,146,669 to £1,491,278)	£1,014,363 (£19,462 to £2,009,264)
NMB £20,000 per QALY (per patient)			£12,163 (£5 to £24,321)			£12,015 (£338 to £23,692)	£148 (−£2208 to £2504)

*LOS* length of stay, *QALYs* quality adjusted life years, *DID* difference-in-difference, *NMB* net monetary benefit

At 10 years, both reconfigurations resulted in more QALYs compared to their Rest of England comparator (Table 4). London resulted in 58 more QALYs at 10 years per 1000 patients at an additional cost of £1,014,363. This is equivalent to an incremental cost effectiveness ratio of £17,452; hence, at a WTP for a QALY gained of £20,000, the changes in London have a higher NMB than the rest of England over the same time period. However, the incremental cost effectiveness ratio is greater than the average cost to produce a QALY in the NHS of £12,936, suggesting that some displacement of services and a net QALY loss may have occurred. At 10 years, the reconfigurations that occurred in GM dominate what happened in England over the same time period in that there are more QALYs (18 QALYs per 1000 patients over 10 years compared to the rest of England) and less costs (−£470,848 per 1000 patients compared to the rest of England over 10 years).

**Table 4:** Mean and 95% confidence intervals (CIs) for costs, mortality and QALYs, at 90 days and 10 years for Greater Manchester (GM) per 1000 patients

	Greater Manchester			England			DID
	Before	After	Difference	Before	After	Difference	
90-day results							
Deaths	127 (103 to 151)	102 (76 to 127)	−25 (−46 to −5)	130 (109 to 152)	103 (84 to 121)	−27 (−33 to −22)	2 (−19 to 23)
LOS	20 (19 to 22)	16 (14 to 17)	−6 (−6 to −4)	19 (18 to 21)	17 (16 to 18)	−3 (−3 to −2)	−2 (−3 to −1)
QALYs	96 (90 to 102)	103 (93 to 113)	7 (−1 to 15)	98 (92 to 104)	103 (97 to 110)	6 (5 to 6)	2 (−7 to 10)
Costs	£5,589,377 (£4,441,146 to £6,737,608)	£5,214,732 (£4,263,891 £6,165,572)	–£374,645 (−£740,668 to –£8622)	£5,435,973 (£4,339,780 to £6,532,166)	£4,905,210 (£3,902,177 to £5,908,243)	–£530,764 (−£653,699 to –£407,828)	£156,119 (−£169,632 to £481,869)
10-year results							
Deaths	426 (360 to 492)	399 (329 to 468)	−27 (−44 to −9)	429 (364 to 494)	406 (333 to 479)	−23 (−51 to 6)	−4 (−32 to 24)
QALYs	2751 (2336 to 3167)	3285 (2906 to 3664)	534 (113 to 954)	2747 (2339 to 3156)	3263 (2882 to 3644)	516 (103 to 929)	18 (−122 to 158)
Costs	£39,444,918 (£34,251,242 to £44,638,594)	£37,087,053 (£26,978,822 to £47,195,284)	–£2,357,865 (−£6,381,868 to £1,666,137)	£39,092,117 (£29,011,793 to £49,172,441)	£37,205,099 (£26,931,764 to –£47,544,760)	–£1,887,018 (−£5,662,138 to £1,888,103)	–£470,848 (−£1,882,646 to £940,951)
NMB £20,000 per QALY (per patient)			£13,033 (£1244 to £24,822)			£12,199 (£674 to £23,723)	£834 (−£1683 to £3350)

*LOS* length of stay, *QALYs* quality adjusted life years, *DID* difference-in-difference, *NMB* net monetary benefit

### Cost-effectiveness acceptability curve

**Fig. 3:**
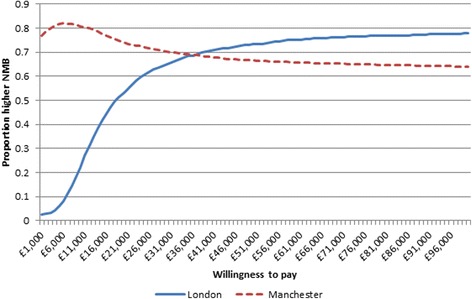
Cost-effectiveness acceptability curve of the probability that the reconfigurations in London and GM resulted in a higher NMB compared to England over the same time period

**Fig. 4:**
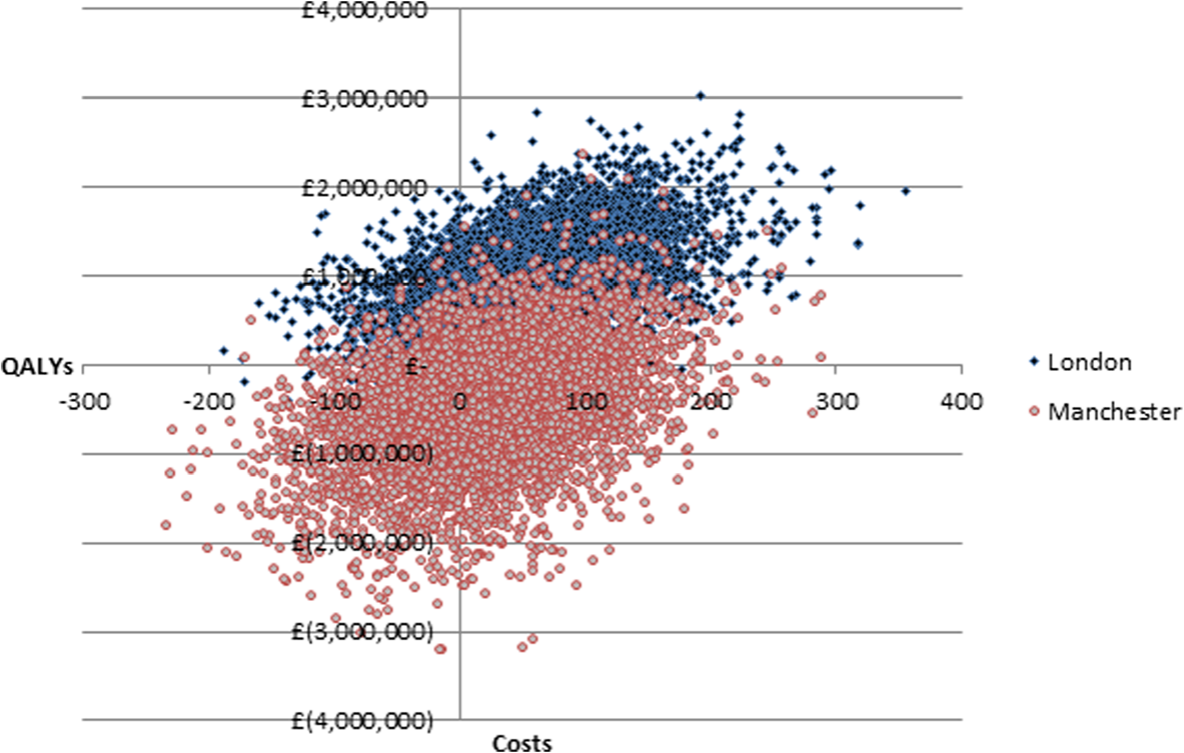
Difference-in-difference cost-effectiveness plane of the adjusted difference in 10-year costs and QALYs between London before and after reconfigurations minus the difference in England over the same time period, and the difference in costs and QALYs in GM compared to England over the same time period

The probability that the changes that occurred in GM as a result of the reconfigurations had a higher NMB than what occurred in England over the same time period peaked at a WTP for a QALY gained of £7000 and 82% probability (Fig. 3). GM had a higher probability of being cost-effective than London at a WTP for a QALY gained of values less than £39,000. GM had a higher probability of being cost-effective at lower values of WTP for a QALY as 35% of iterations of the difference in costs and QALYs in GM compared to England over the same time period fell in the south-west quadrant of the cost-effectiveness plane (Fig. 4); thus, the changes in GM cost less than in the rest of England but there was only a 60% chance that there was also a reduction in mortality compared to the rest of England. For London, the majority of iterations of the model (80%) fell in the north-east quadrant for London, where London cost more but also had a higher probability that there was an improvement in mortality (81%).

### Sensitivity analyses

The results of the sensitivity analyses are reported in Additional file 1: Tables S6 and S7. None of the sensitivity analyses changed the conclusions drawn as a result of the analysis, other than the observation that, once the cost of a HASU increased by 50% per night, the London reconfigurations were no longer cost-effective at a WTP of £20,000 per QALY. If it was assumed that improvements in functioning (Barthel index) only occurred in London and GM as a result of the reconfiguration but not in the rest of England, there was a 97% chance in both London and GM that the reconfigurations be cost-effective at 10 years at a WTP of £20,000 for a QALY (Additional file 1: Figure S2).

### Costs of implementation

In addition to the funding for the increased tariff, additional one-off financial investments were made to bring about the reconfigurations in London and GM. It was estimated that an investment of £9 million was made in London to meet the requirements for the new HASUs and stroke units, covering capital, equipment and premises refurbishment [21]. The cost of the tariff uplift has been included in the analysis already as the increased cost per bed day in a HASU. In 2014/2015, there were 6641 people admitted to hospital with a primary diagnosis of stroke in London [20]. Assuming that the London model will continue to be in place for the next 10 years, and a constant rate of admission for stroke, the cost of implementation works out to £136 per patient admitted with stroke over this period. In GM, it was estimated that an initial investment of £2.79 million was required for similar items [22]. In 2014/2015, there were 3732 people admitted to hospital with a primary diagnosis of stroke in GM [20]. Making the same assumptions as for London, the cost per patient of the reconfigurations in GM was £75 per patient admitted. Other costs are likely to have been incurred in both London and GM to pay for research to identify the optimal configuration of services, and to consult staff and public, but evidence of these costs is not available.

### QALY production for a fixed budget of £40 million

The results of the analysis comparing total strokes treated and total QALYs at 1, 5 and 10 years for a fixed yearly budget of £40 million are reported in Table 5. We estimated 75 additional QALYs over 10 years as the result of the centralisation of GM acute stroke care compared to changes that occurred in the rest of England for the same time period for a fixed yearly budget of £40 million. Centralisation of London services may have resulted in a net loss of 720 QALYs over 10 years. The lower number of estimated QALYs is a result of a lower volume of patients that may be treated for the fixed budget.

**Table 5:** Total number of strokes treated and total QALYs for a fixed yearly budget of £40 million

	Before	After	Difference	Before	After	Difference	DID
	London			England			
Number of strokes treated per year	4001	3522	−480	3959	3736	−222	−257
QALYs 1 year	1560	1608	49	1500	1653	152	−104
QALYs 5 years	8342	8943	602	7946	9026	1080	−479
QALYs 10 years	13,047	14,067	1020	12,382	14,122	1740	−720
	GM			England			
Number of strokes treated per year	3947	3716	−231	4017	3771	−246	15
QALYs 1 year	1470	1619	150	1494	1659	165	−15
QALYs 5 years	7782	8966	1184	7900	9054	1154	30
QALYs 10 years	12,122	14,056	1934	12,304	14,164	1860	75

*GM* Greater Manchester, *QALYs* quality adjusted life years, *DID* difference-in-difference

## Discussion

The implementation of major system change in both London and GM models resulted in a NMB compared to changes that occurred over the same time period elsewhere in England at 10 years at a threshold of £20,000–£30,000 for a QALY. The GM model was cost saving at 10 years, but did not result in the same health benefits as the London model; hence, for higher values of WTP for a QALY, London had a higher probability of being cost-effective.

If the value for money of the centralisation of acute care is tested in the context of PBMA using a finite budget of £40 million per year (approximate budget required for 4000 stroke admissions per year) and choosing the model that produces the largest number of QALYs, the GM model may be preferred based on the QALYs produced at 5 and 10 years. Over time, the cost of treating strokes in metropolitan areas in England has increased everywhere, but clinical outcomes have also improved. As a result, even though fewer people can be treated for a stroke within the same budget envelope, the resulting total QALYs (health benefits) may still be greater.

### Reporting the results of economic evaluations to decision-makers

The aim of this study was to present the results of the cost-utility analysis in two contrasting ways, namely (1) as cost per QALY in line with NICE recommendations and (2) as a PBMA to reflect a potential real-world scenario. Within the English NHS, decision-making based on economic evaluations predominately occurs as part of NICE technology assessments and guidelines [23]. As a result, most cost-effectiveness analyses are conducted using the NICE reference case as their template for their methodology and reporting with little consideration given to whether this is helpful to decision-makers at regional and local levels [23]. Previous research has identified that improved clinical outcomes play a key role in the decision to implement change [23, 24]. Clinicians in particular prefer that clinical effectiveness is given more weight, arguing for the needs of the patients [25]. Both the results of the NMB and the PBMA would recommend that regions implement the GM model for acute stroke care reconfiguration. However, the fact that GM changed aspects of its delivery of stroke care following evidence of its lack of clinical impact is a reflection of the important role that clinical outcomes play. Indeed, this analysis is only one component of a wider piece of work looking at how service reconfiguration comes about in acute stroke services, with a key emphasis on clinical and organisational outcomes [2, 4, 5, 24].

When costs are considered at local and regional levels, these tend to relate to identifying additional resources for staffing and the costs of new equipment. Potential disinvestments in other services are rarely considered. Where value for money is mentioned, it is more commonly used to refer to cost-savings, and not the cost per outcome gained more commonly presented in economic evaluations [23]. Regardless of the potential benefits of centralisation of services one of the greatest challenges for commissioners is identifying funding for the initial upfront cost of reconfiguring services. However, previous evaluations of major system change have failed to address the economic implications of different implementation strategies [7, 24, 26]. As reported in our other work [24, 27], this may be one of the greatest barriers to major system changes. To help decision-makers, we have provided as much information as possible on the potential costs of implementation. However, more work is required to identify to what extent these represent additional resource requirements in regards to additional staffing and equipment.

A systematic review of economic evaluations of centralisation of specialist services identified that most economic evaluations lack information that is sufficiently detailed and transparent to be useful to decision-makers [7]. As part of our work, we consulted with decision-makers responsible for decisions about stroke service reconfiguration, including having them as members on steering groups, as part of our qualitative work, informal interviews and correspondence [2]. This was done to ensure that we present information that is useful to decision-makers and has been a driver in our decision to report the results as a PBMA. Reporting results over different time horizons and for a range of outcomes, not just QALYs, has also been recommended when reporting results for decision-makers [7, 28]. Previous research has suggested that decision-makers rarely use QALYs, preferring hard clinical outcomes instead as they find them easier to understand [23, 28]. However, we have chosen to keep with QALYs given these can capture both mortality and morbidity – an important consideration in stroke care, which aims to improve functioning in addition to extending life. Nevertheless, we have continued to report mortality and LOS alongside QALYs given that decision-makers find these outcomes easier to understand than QALYs and consider them important outcomes in stroke care.

Another important consideration for reporting the results is their generalisability and clear communication of limitations [7, 23]. We have tried to make the results generalisable by adjusting for key factors that are likely to differ across regions in our analysis, for example, age and type of stroke. As part of the PBMA, we chose a population size that is representative of the average metropolitan area in England. However, the analysis is based on major system change in two metropolitan areas. A key limitation of this analysis is that the results are not applicable to rural areas, which may face different challenges in the centralisation of acute stroke services. In particular, rural areas will need to address the issue of greater travel times as a result of reconfiguration and may require different solutions to address these, for example, telemedicine, with different costs to consider in bringing about these changes [4].

### Strengths and weaknesses

The key strength of this analysis is that it provides information to policy-makers about the real-world implications of the changes made and hence can inform policy decisions about major system changes in the future. It contains information on the upfront costs of implementation, as well as ongoing costs, and presents the results both in regards to clinical effectiveness, but also using PBMA and within the context of a fixed budget.

A key weakness is that the results are based on observational, routinely collected hospital data; most notable are coding errors. For example, in one London hospital, 3% of 1300 stroke patients had a discharge destination code for discharge to a mental health hospital, compared to 0.13% for hospitals across all of England. It is unlikely that such a large percentage of patients were being discharged to this location; instead, it is more likely that the wrong code had been used and they were being discharged elsewhere. There is no way to know where and hence the only option was to remove the data prior to estimating discharge destination. It is likely the dataset contains other errors in relation to discharge destination. However, it was assumed that those errors would occur equally at random across the three regions. It is also hard to know how ‘stroke mimics’, patients presenting with the stroke-like symptoms but who do not have a final diagnosis of stroke, appear in the data. They may be screened out of the datasets as not having stroke even though they have a stroke unit admission, or potentially the final diagnosis is missing from our dataset. It is hard to know from the data we have what proportion are mimics and what impact they have had on the cost and outcomes for the care pathway.

The retrospective nature of the study also meant that limited information was available on the true cost of implementation. Although data could be obtained from reports and interviews with stakeholders, some of the knowledge of the true cost of implementation would have been lost. This knowledge and the importance of collecting in-depth data on the cost of implementation has assisted with the design of other studies [29].

It is important to note that the values reported in the model for LOS and mortality are values adjusted for differences between patients within the regions with regards to age, ethnicity, sex and co-morbidities. As a result, they do not represent true values per 1000 or 4000 patients but adjusted values to allow for comparison between areas over the same period of time. One of the key weaknesses in the model is that South London is the only area where data were available on what impact the changes had on functioning using the Barthel index. Instead, we had to make the conservative estimate that the same improvement in functioning was seen throughout the whole country over the same time period. If we assume that instead the improvements are the direct result of the reconfigurations, the sensitivity analysis showed that the London and GM models have a significantly higher probability of being cost-effective.

A further limitation of the study is that we were unable to calculate a ward-specific probability of being discharged. As a result, some wards may have had higher or lower LOS than in real life. However, the main ward this will have had an impact on is the intensive therapy unit (ITU), as it has a higher cost and lower utility score than other wards. Nevertheless, there were only a small percentage of people admitted to ITU and hence it is unlikely to have had a significant impact on the results. In one of the sensitivity analyses, we increased the percentage of people admitted to ITU with little impact on the results.

## Conclusions

Economic factors are a significant determinant in the decision to implement major system change [27], but the results of economic evaluations of centralisation are rarely reported in a way that is helpful to decision-makers [7]. How the results of economic evaluations are reported may have a significant impact on the decision to implement change and how a service should be reconfigured. Although a centralised model of stroke care across an entire metropolitan area has a high probability of being a cost-effective way to improve outcomes for stroke patients at the threshold of £30,000 per QALY, what this model of stroke care should look like is dependent on the priorities of a decision-maker. If the aim of the payer is to maximise clinical outcomes with no consideration of budget, then the London model may be the preferable one. In the context of fixed health budgets, there is the potential that the additional cost of centralisation may displace benefits of other services that potentially produce more QALYs for a lower cost. In the case of GM, it may be that the reconfigurations resulted in a net health benefit to the health economy as a result of the reduced cost per stroke even though they did not result in additional clinical improvements.

## Additional file

Additional file 1:  Supporting figures, tables and methods for acute stroke cost-utility analysis. (DOCX 81 kb)

## Abbreviations

GM: Greater Manchester
HASU: hyper-acute stroke unit
HES: Hospital Episode Statistics
ITU: intensive therapy unit
LOS: length of stay
NHS: National Health Service
NICE: National Institute for Health and Care Excellence
NMB: net monetary benefit
PBMA: programme budgeting and marginal analysis
QALY: quality adjusted life years
SINAP: Stroke Improvement National Audit Programme
SLSR: South London Stroke Register
WTP: willingness to pay
