# Supplementary material for: The potential role of cost-utility analysis in the decision to implement major system change in acute stroke services in metropolitan areas in England
Source: Health Res Policy Syst. 2018 Mar 14;16:23. doi: 10.1186/s12961-018-0301-5 (PMC5852958; doi:10.1186/s12961-018-0301-5)
Supplement: Supplementary file 1 — Supporting figures, tables and methods for acute stroke cost-utility analysis. (DOCX 81 kb) [file 12961_2018_301_MOESM1_ESM.docx]

Additional file 1

Figure S1: 10 year Markov model structure

**Institutional**

**care**

**Recurrent stroke**

**Dead**

**Home**

**(function level: independent; mild; moderate; severe; very severe)**

**Day 90**

Table S1: Coefficients for calculating daily probability of time from admission until death

| **Variable** | **Constant (SE)** | **β Coefficient Before (SE)** | **β Coefficient After (SE)** |
| --- | --- | --- | --- |
| **London 72 Hours Mortality** |  |  |  |
| London 72 hours- Hospital | -2.547443 (0.1536) | -0.1304453 (0.0892) | -0.3388409 (0.1191) |
| London 72 hours – Home Before | -1.112563 (0.0291) | -5.498865 (0.206321) |  |
| London 72 hours – Home After | -0.9000484 (0.044) |  | -6.268149 (0.33579) |
| London 72 hours – Community Hospital Before | -1.112563 (0.0291) | -3.914968 (0.238261) |  |
| London 72 hours – Community Hospital After | -0.9000484 (0.044) |  | -5.442073 (0.57907) |
| **London 30 days Mortality** |  |  |  |
| London 30 days- Hospital | -2.196218 (0.12067) | -0.1400739 (0.05099) | -0.4069234 (0.0853) |
| London 30 days – Home Before | 1.434057 (0.045317) | -6.052406 (0.1111684) |  |
| London 30 days – Home After | 1.455995 (0.051168) |  | -6.040282 (0.11063) |
| London 30 days – Nursing Home Before | 1.434057 (0.045317) | -5.697617 (0.255799) |  |
| London 30 days – Nursing Home After | 1.455995 (0.051168) |  | -6.091694 (0.32185) |
| London 30 days – Community Hospital Before | 1.434057 (0.045317) | -4.8455572 (0.158142) |  |
| London 30 days – Community Hospital After | 1.455995 (0.051168) |  | -4.442539 (0.12421) |
| **London 90 days Mortality** |  |  |  |
| London 90 days- Hospital | -2.116191 (0.11408) | -0.1210432 (0.044904) | -0.4217429 (0.0639) |
| London 90 days – Home Before | 3.735711 (0.118429) | -7.01472 (0.1299083) |  |
| London 90 days – Home After | 3.272364 (0.106797) |  | -6.622667 (0.11198) |
| London 90 days – Nursing Home Before | 3.735711 (0.118429) | -5.945526 (0.1541422) |  |
| London 90 days – Nursing Home After | 3.272364 (0.106797) |  | -5.449472 (0.14801) |
| London 90 days – Community Hospital Before | 3.735711 (0.118429) | -6.185021 (0.1540291) |  |
| London 90 days – Community Hospital After | 3.272364 (0.106797) |  | -5.326356 (0.13123) |
| **England (vs London) 72 Hours Mortality** |  |  |  |
| England 72 hours- Hospital | -2.547443 (0.1536) | 0 | -0.11557 (0.028474) |
| England 72 hours – Home Before | -0.8274124 (0.0127) | -5.915824 (0.102904) |  |
| England 72 hours – Home After | -0.7733679 (0.0150) |  | -6.168171 (0.11886) |
| England 72 hours – Nursing Home Before | -0.8274124 (0.0127) | -7.27151 (0.5002305) |  |
| England 72 hours – Nursing Home After | -0.7733679 (0.0150) |  | -7.121694 (0.50031) |
| England 72 hours – Community Hospital Before | -0.8274124 (0.0127) | -4.699328 (0.1723066) |  |
| England 72 hours – Community Hospital After | -0.7733679 (0.0150) |  | -5.350084 (0.25072) |
| **England (vs London) 30 days Mortality** |  |  |  |
| England 30 days- Hospital | -2.196218 (0.12067) | 0 | -0.10559 (0.025677) |
| England 30 days – Home Before | 1.595699 (0.015582) | -5.961341 (0.0351102) |  |
| England 30 days – Home After | 1.790226 (0.019933) |  | -6.014796 (0.03662) |
| England 30 days – Nursing Home Before | 1.595699 (0.015582) | -5.66156 (0.0696054) |  |
| England 30 days – Nursing Home After | 1.790226 (0.019933) |  | -5.425521 (0.06545) |
| England 30 days – Community Hospital Before | 1.595699 (0.015582) | -4.592778 (0.0530674) |  |
| England 30 days – Community Hospital After | 1.790226 (0.019933) |  | -5.02211 (0.064315) |
| **England (vs London) 90 days Mortality** |  |  |  |
| England 90 days- Hospital | -2.116191 (0.11408) | 0 | -0.22544 (0.019982) |
| England 90 days – Home Before | 4.030386 (0.044542) | -7.16257 (0.0478558) |  |
| England 90 days – Home After | 4.325614 (0.061488) |  | -7.408664 (0.33580) |
| England 90 days – Nursing Home Before | 4.030386 (0.044542) | -6.149743 (0.0526994) |  |
| England 90 days – Nursing Home After | 4.325614 (0.061488) |  | -6.262616 (0.06802) |
| England 90 days – Community Hospital Before | 4.030386 (0.044542) | -5.865234 (0.0544597) |  |
| England 90 days – Community Hospital After | 4.325614 (0.061488) |  | -6.268872 (0.0709) |
| **GM 72 Hours Mortality** |  |  |  |
| GM 72 hours- Hospital | -2.421833 (0.14417) | -0.0163415 (0.072725) | -0.1513003 (0.1442) |
| GM 72 hours – Home Before | -0.8689113 (0.0747) | -6.182511 (0.7113363) |  |
| GM 72 hours – Home After | -0.7285307 (0.0561) |  | -7.156792 (0.70946) |
| GM 72 hours – Community Hospital Before | -0.8689113 (0.0747) | -3.998623 (1.006607) |  |
| GM 72 hours – Community Hospital After | -0.7285307 (0.0561) |  | -5.517576 (1.00254) |
| **GM 30 days Mortality** |  |  |  |
| GM 30 days- Hospital | -2.015169 (0.11623) | -0.0612502 (0.056943) | -0.2228965 (0.0977) |
| GM 30 days – Home Before | 1.291925 (0.082786) | -6.084666 (0.2447883) |  |
| GM 30 days – Home After | 1.792565 (0.075070) |  | -6.185732 (0.14564) |
| GM 30 days – Nursing Home Before | 1.291925 (0.082786) | -6.452402 (0.713927) |  |
| GM 30 days – Nursing Home After | 1.792565 (0.075070) |  | -5.948318 (0.32742) |
| GM 30 days – Community Hospital Before | 1.291925 (0.082786) | -3.898721 (0.3551919) |  |
| GM 30 days – Community Hospital After | 1.792565 (0.075070) |  | -4.118781 (0.17175) |
| **GM 90 days Mortality** |  |  |  |
| GM 90 days- Hospital | -1.952976 (0.11159) | -0.0042823 (0.063693) | -0.2941545 (0.0843) |
| GM 90 days – Home Before | 3.471728 (0.199914) | -6.677631 (0.2263131) |  |
| GM 90 days – Home After | 3.615176 (0.164399) |  | -6.821608 (0.17903) |
| GM 90 days – Nursing Home Before | 3.471728 (0.199914) | -5.92642 (0.2800679) |  |
| GM 90 days – Nursing Home After | 3.615176 (0.164399) |  | -5.604104 (0.20396) |
| GM 90 days – Community Hospital Before | 3.471728 (0.199914) | -5.127685 (0.3104291) |  |
| GM 90 days – Community Hospital After | 3.615176 (0.164399) |  | -5.373488 (0.20603) |
| **England (vs GM) 72 Hours Mortality** |  |  |  |
| England 72 hours- Hospital | -2.421833 (0.14417) | 0 | -0.16135 (0.031914) |
| England 72 hours – Home Before | -0.8274124 (0.0127) | -5.915824 (0.102904) |  |
| England 72 hours – Home After | -0.7733679 (0.0150) |  | -6.168171 (0.11886) |
| England 72 hours – Nursing Home Before | -0.8274124 (0.0127) | -7.27151 (0.5002305) |  |
| England 72 hours – Nursing Home After | -0.7733679 (0.0150) |  | -7.121694 (0.50031) |
| England 72 hours – Community Hospital Before | -0.8274124 (0.0127) | -4.699328 (0.1723066) |  |
| England 72 hours – Community Hospital After | -0.7733679 (0.0150) |  | -5.350084 (0.25072) |
| **England (vs GM) 30 days Mortality** |  |  |  |
| England 30 days- Hospital | -2.015169 (0.11623) | 0 | -0.26858 (0.0253) |
| England 30 days – Home Before | 1.595699 (0.015582) | -5.961341 (0.0351102) |  |
| England 30 days – Home After | 1.790226 (0.019933) |  | -6.014796 (0.03662) |
| England 30 days – Nursing Home Before | 1.595699 (0.015582) | -5.66156 (0.0696054) |  |
| England 30 days – Nursing Home After | 1.790226 (0.019933) |  | -5.425521 (0.06545) |
| England 30 days – Community Hospital Before | 1.595699 (0.015582) | -4.592778 (0.0530674) |  |
| England 30 days – Community Hospital After | 1.790226 (0.019933) |  | -5.02211 (0.064315) |
| **England (vs GM) 90 days Mortality** |  |  |  |
| England 90 days- Hospital | -1.952976 (0.11159) | 0 | -0.30497 (0.023264) |
| England 90 days – Home Before | 4.030386 (0.044542) | -7.16257 (0.0478558) |  |
| England 90 days – Home After | 4.325614 (0.061488) |  | -7.408664 (0.33580) |
| England 90 days – Nursing Home Before | 4.030386 (0.044542) | -6.149743 (0.0526994) |  |
| England 90 days – Nursing Home After | 4.325614 (0.061488) |  | -6.262616 (0.06802) |
| England 90 days – Community Hospital Before | 4.030386 (0.044542) | -5.865234 (0.0544597) |  |
| England 90 days – Community Hospital After | 4.325614 (0.061488) |  | -6.268872 (0.0709) |

SE= standard error GM= Greater Manchester

Table S2: Coefficients for calculating daily probability of time from hospital admission to discharge

| **Variable** | **Constant (SE)** | **β Coefficient Before (SE)** | **β Coefficient After (SE)** | **Gamma** |
| --- | --- | --- | --- | --- |
| **London** |  |  |  |  |
| London – daily probability of discharge home | -2.64098 (0.030461) | 0.045712 (0.009146) | 0.214972 (0.00943) | -0.10706 (0.001542) |
| London – discharge to care home (publicly funded) | -2.64098 (0.030461) | -0.973  (0.0383368) | -0.98968  (0.04183) | -0.10706 (0.001542) |
| London – discharge to care home (privately funded) | -2.64098 (0.030461) | -1.0295  (0.0576309) | -1.07139  (0.059219) | -0.10706 (0.001542) |
| London – discharge to community hospital | -2.64098 (0.030461) | -0.5085  (0.0293949 | -0.1767  (0.027445) | -0.10706 (0.001542) |
| **England (vs London)** |  |  |  |  |
| England – daily probability of discharge home | -2.64098 (0.030461) | 0 | 0.155154  (0.004678) | -0.10706 (0.001542) |
| England – discharge to care home (publicly funded) | -2.64098 (0.030461) | -0.87083  (0.01323) | -0.89047  (0.014033) | -0.10706 (0.001542) |
| England – discharge to care home (privately funded) | -2.64098 (0.030461) | -0.99054  (0.013744) | -1.06343  (0.015379) | -0.10706 (0.001542) |
| England – discharge to community hospital | -2.64098 (0.030461) | -0.29652  (0.011769) | -0.36854  (0.012613) | -0.10706 (0.001542) |
| **GM** |  |  |  |  |
| GM – daily probability of discharge home | -2.64936  (0.034407) | -0.06769  (0.012846) | 0.1702  (0.018182) | -0.10741  (0.001654) |
| GM – discharge to care home (publicly funded) | -2.64936  (0.034407) | -0.8935  (0.074893) | -0.91537  (0.051917) | -0.10741  (0.001654) |
| GM – discharge to care home (privately funded) | -2.64936  (0.034407) | -0.77431  (0.092258) | -0.99686  (0.071256) | -0.10741  (0.001654) |
| GM – discharge to community hospital | -2.64936  (0.034407) | -0.15751  (0.091618) | 0.319658  (0.050619) | -0.10741  (0.001654) |
| **England (vs GM)** |  |  |  |  |
| England – daily probability of discharge home | -2.64936  (0.034407) | 0 | 0.173084  (0.006077) | -0.10741  (0.001654) |
| England – discharge to care home (publicly funded) | -2.64936  (0.034407) | -0.87083  (0.01323) | -0.89047  (0.014033) | -0.10741  (0.001654) |
| England – discharge to public care home | -2.64936  (0.034407) | -0.99054  (0.013744) | -1.06343  (0.015379) | -0.10741  (0.001654) |
| England – discharge to community hospital | -2.64936  (0.034407) | -0.29652  (0.011769) | -0.36854  (0.012613) | -0.10741  (0.001654) |

SE= Standard Error GM= Greater Manchester

Table S3: Percentage of patients discharged to each discharge destination

| Analysis | **Home**  **% (n)** | **Care Home (publicly funded)**  **% (n)** | **Care Home (privately funded)**  **% (n)** | **Community hospital**  **%(n)** |
| --- | --- | --- | --- | --- |
| London Before | 80% (9 848) | 6% (746) | 3% (313) | 11% (1 363) |
| London After | 80% (10 023) | 5% (622) | 2% (296) | 13% (1 635) |
| Manchester Before | 83% (2 262) | 7% (198) | 5% (125) | 5% (130) |
| Manchester After | 83% (5 220) | 6% (407) | 3% (207) | 8% (511) |
| England Before | 80% (76 669) | 6% (6 261) | 6% (5 774) | 8% (8 274) |
| England After | 80% (74 546) | 6% (5 886) | 5% (4 874) | 8% (7 319) |

Table S4: Ward of admission

| Analysis | **Stroke Unit**  **%** | **Medical Assessment Unit**  **%** | **General medical**  **%** | **ITU/CCU**  **%** | **Other** |
| --- | --- | --- | --- | --- | --- |
| London Before | 27% | 59% | 6% | 2% | 5% |
| London After | 87% | 7% | 2% | 2% | 2% |
| Manchester Before | 16% | 80% | 1% | 1% | 2% |
| Manchester After | 60% | 34% | 2% | 2% | 3% |
| England Before | 15% | 78% | 3% | 2% | 2% |
| England After | 68% | 26% | 2% | 1% | 3% |

ITU= Intensive treatment unit; CCU=Critical care unit

Table S4: Values for 10 year model.

| **Movement from** | **Movement to** | **Transition probability** | **Time period/ variable type** | **Source** |
| --- | --- | --- | --- | --- |
| **London Before** |  |  |  |  |
| Home | Residential care | 0.014 | 90 days to 1 year | SLSR |
| Home | Residential care | 0.003 | 1 year to 10 years | SLSR |
| Home | Recurrent stroke | 0.022 | Constant | HES |
| Home | Recurrent stroke | 0.585 | Gamma | HES |
| Home | Dead | 0.042 | Constant | HES |
| Home | Dead | 0.459 | Gamma | HES |
| Residential care | Recurrent stroke | 0.018 | Constant | HES |
| Residential care | Recurrent stroke | 0.585 | Gamma | HES |
| Residential care | Dead | 0.085 | Up to end of year 10 | Gordon 2014 |
| Recurrent stroke | Home: BI = 20 | 0.300 | Up to end of year 10 | SLSR |
| Recurrent stroke | Home: BI = 15-19 | 0.244 | Up to end of year 10 | SLSR |
| Recurrent stroke | Home: BI = 10-14 | 0.131 | Up to end of year 10 | SLSR |
| Recurrent stroke | Home: BI = 5-9 | 0.075 | Up to end of year 10 | SLSR |
| Recurrent stroke | Home: BI = 0-4 | 0.094 | Up to end of year 10 | SLSR |
| Recurrent stroke | Residential care | 0.057 | Up to end of year 10 | From 90 day model |
| Recurrent stroke | Dead | 0.100 | Up to end of year 10 | From 90 day model |
| Recurrent stroke | Hospital 90+days | 0.115 | Up to end of year 10 | From 90 day model |
| Hospital | Recurrent stroke | 0.048 | Constant | HES |
| Hospital | Recurrent stroke | 0.585 | Gamma | HES |
| **London After** |  |  |  |  |
| Home | Residential care | 0.014 | 90 days to 1 year | SLSR |
| Home | Residential care | 0.003 | 1 year to 10 years | SLSR |
| Home | Recurrent stroke | 0.021 | Constant | HES |
| Home | Recurrent stroke | 0.585 | Gamma | HES |
| Home | Dead | 0.040 | Constant | HES |
| Home | Dead | 0.459 | Gamma | HES |
| Residential care | Recurrent stroke | 0.018 | Constant | HES |
| Residential care | Recurrent stroke | 0.585 | Gamma | HES |
| Residential care | Dead | 0.085 | Up to end of year 10 | Gordon 2014 |
| Recurrent stroke | Home: BI = 20 | 0.474 | Up to end of year 10 | SLSR |
| Recurrent stroke | Home: BI = 15-19 | 0.246 | Up to end of year 10 | SLSR |
| Recurrent stroke | Home: BI = 10-14 | 0.046 | Up to end of year 10 | SLSR |
| Recurrent stroke | Home: BI = 5-9 | 0.055 | Up to end of year 10 | SLSR |
| Recurrent stroke | Home: BI = 0-4 | 0.055 | Up to end of year 10 | SLSR |
| Recurrent stroke | Residential care | 0.052 | Up to end of year 10 | From 90 day model |
| Recurrent stroke | Dead | 0.074 | Up to end of year 10 | From 90 day model |
| Recurrent stroke | Hospital 90+days | 0.115 | Up to end of year 10 | From 90 day model |
| Hospital | Recurrent stroke | 0.048 | Constant | HES |
| Hospital | Recurrent stroke | 0.585 | Gamma | HES |
| **Manchester Before** |  |  |  |  |
| Home | Residential care | 0.014 | 90 days to 1 year | SLSR |
| Home | Residential care | 0.003 | 1 year to 10 years | SLSR |
| Home | Recurrent stroke | 0.022 | Constant | HES |
| Home | Recurrent stroke | 0.585 | Gamma | HES |
| Home | Dead | 0.045 | Constant | HES |
| Home | Dead | 0.459 | Gamma | HES |
| Residential care | Recurrent stroke | 0.018 | Constant | HES |
| Residential care | Recurrent stroke | 0.585 | Gamma | HES |
| Residential care | Dead | 0.085 | Up to end of year 10 | Gordon 2014 |
| Recurrent stroke | Home: BI = 20 | 0.285 | Up to end of year 10 | SLSR |
| Recurrent stroke | Home: BI = 15-19 | 0.231 | Up to end of year 10 | SLSR |
| Recurrent stroke | Home: BI = 10-14 | 0.125 | Up to end of year 10 | SLSR |
| Recurrent stroke | Home: BI = 5-9 | 0.071 | Up to end of year 10 | SLSR |
| Recurrent stroke | Home: BI = 0-4 | 0.089 | Up to end of year 10 | SLSR |
| Recurrent stroke | Residential care | 0.072 | Up to end of year 10 | From 90 day model |
| Recurrent stroke | Dead | 0.127 | Up to end of year 10 | From 90 day model |
| Recurrent stroke | Hospital 90+days | 0.107 | Up to end of year 10 | From 90 day model |
| Hospital | Recurrent stroke | 0.048 | Constant | HES |
| Hospital | Recurrent stroke | 0.585 | Gamma | HES |
| **Manchester After** |  |  |  |  |
| Home | Residential care | 0.014 | 90 days to 1 year | SLSR |
| Home | Residential care | 0.003 | 1 year to 10 years | SLSR |
| Home | Recurrent stroke | 0.024 | Constant | HES |
| Home | Recurrent stroke | 0.585 | Gamma | HES |
| Home | Dead | 0.043 | Constant | HES |
| Home | Dead | 0.459 | Gamma | HES |
| Residential care | Recurrent stroke | 0.018 | Constant | HES |
| Residential care | Recurrent stroke | 0.585 | Gamma | HES |
| Residential care | Dead | 0.085 | Up to end of year 10 | Gordon 2014 |
| Recurrent stroke | Home: BI = 20 | 0.455 | Up to end of year 10 | SLSR |
| Recurrent stroke | Home: BI = 15-19 | 0.236 | Up to end of year 10 | SLSR |
| Recurrent stroke | Home: BI = 10-14 | 0.044 | Up to end of year 10 | SLSR |
| Recurrent stroke | Home: BI = 5-9 | 0.053 | Up to end of year 10 | SLSR |
| Recurrent stroke | Home: BI = 0-4 | 0.053 | Up to end of year 10 | SLSR |
| Recurrent stroke | Residential care | 0.059 | Up to end of year 10 | From 90 day model |
| Recurrent stroke | Dead | 0.101 | Up to end of year 10 | From 90 day model |
| Recurrent stroke | Hospital 90+days | 0.107 | Up to end of year 10 | From 90 day model |
| Hospital | Recurrent stroke | 0.048 | Constant | HES |
| Hospital | Recurrent stroke | 0.585 | Gamma | HES |
| **England Before** |  |  |  |  |
| Home | Residential care | 0.014 | 90 days to 1 year | SLSR |
| Home | Residential care | 0.003 | 1 year to 10 years | SLSR |
| Home | Recurrent stroke | 0.022 | Constant | HES |
| Home | Recurrent stroke | 0.585 | Gamma | HES |
| Home | Dead | 0.045 | Constant | HES |
| Home | Dead | 0.459 | Gamma | HES |
| Residential care | Recurrent stroke | 0.018 | Constant | HES |
| Residential care | Recurrent stroke | 0.585 | Gamma | HES |
| Residential care | Dead | 0.085 | Up to end of year 10 | Gordon 2014 |
| Recurrent stroke | Home: BI = 20 | 0.290 | Up to end of year 10 | SLSR |
| Recurrent stroke | Home: BI = 15-19 | 0.236 | Up to end of year 10 | SLSR |
| Recurrent stroke | Home: BI = 10-14 | 0.127 | Up to end of year 10 | SLSR |
| Recurrent stroke | Home: BI = 5-9 | 0.073 | Up to end of year 10 | SLSR |
| Recurrent stroke | Home: BI = 0-4 | 0.091 | Up to end of year 10 | SLSR |
| Recurrent stroke | Residential care | 0.070 | Up to end of year 10 | From 90 day model |
| Recurrent stroke | Dead | 0.113 | Up to end of year 10 | From 90 day model |
| Recurrent stroke | Hospital 90+days | 0.065 | Up to end of year 10 | From 90 day model |
| Hospital | Recurrent stroke | 0.048 | Constant | HES |
| Hospital | Recurrent stroke | 0.585 | Gamma | HES |
| **England After** |  |  |  |  |
| Home | Residential care | 0.014 | 90 days to 1 year | SLSR |
| Home | Residential care | 0.003 | 1 year to 10 years | SLSR |
| Home | Recurrent stroke | 0.021 | Constant | HES |
| Home | Recurrent stroke | 0.585 | Gamma | HES |
| Home | Dead | 0.044 | Constant | HES |
| Home | Dead | 0.459 | Gamma | HES |
| Residential care | Recurrent stroke | 0.018 | Constant | HES |
| Residential care | Recurrent stroke | 0.585 | Gamma | HES |
| Residential care | Dead | 0.085 | Up to end of year 10 | Gordon 2014 |
| Recurrent stroke | Home: BI = 20 | 0.452 | Up to end of year 10 | SLSR |
| Recurrent stroke | Home: BI = 15-19 | 0.235 | Up to end of year 10 | SLSR |
| Recurrent stroke | Home: BI = 10-14 | 0.043 | Up to end of year 10 | SLSR |
| Recurrent stroke | Home: BI = 5-9 | 0.052 | Up to end of year 10 | SLSR |
| Recurrent stroke | Home: BI = 0-4 | 0.052 | Up to end of year 10 | SLSR |
| Recurrent stroke | Residential care | 0.069 | Up to end of year 10 | From 90 day model |
| Recurrent stroke | Dead | 0.096 | Up to end of year 10 | From 90 day model |
| Recurrent stroke | Hospital 90+days | 0.065 | Up to end of year 10 | From 90 day model |
| Hospital | Recurrent stroke | 0.048 | Constant | HES |
| Hospital | Recurrent stroke | 0.585 | Gamma | HES |

* BI= Barthel Index

Table S5: Utility values

| **Location** | **Mean** | **SE** |
| --- | --- | --- |
| Stroke Unit | 0.24 | 0.027932 |
| Medical ward/General ward | 0.266452 | 0.041781 |
| ITU/CCU | 0.01725 | 0.078934 |
| Other | 0.272308 | 0.102945 |
| Community Hospital | 0.158667 | 0.062064 |
| Nursing Home (public or private) | 0.128947 | 0.056194 |
| Home | 0.557932 | 0.015597 |
| Dead | 0 |  |
| Discharge Other | 0.324857 | 0.112742 |
| Home: BI = 20 | 0.69 | 0.025 |
| Home: BI = 15-19 | 0.60 | 0.010121 |
| Home: BI = 10-14 | 0.37 | 0.024084 |
| Home: BI = 5-9 | 0.10 | 0.040107 |
| Home: BI = 0-4 | -0.06 | 0.025793 |
| Residential Care | 0.09 | 0.025 |

* BI= Barthel Index; ITU=Intensive Therapy Unit; CCU= Coronary Care Unit

Methods S1:

As the 90 day model is made up of 90 one day transitions, the probability of death at each day was then converted to a one day rate using the formula of the log of one minus the exponential of the constant plus the coefficient for the respective model divided by the relative time for each mortality point. This was carried out for zero days to three days, three days to 30 days and 30 days to 90 days. The same daily probability of death was applied to all the patients in hospital regardless of which ward they were on; only time after admission to hospital determined the probability of death for patients in hospital. This was because HES data do not contain information on which ward a patient is in to allow a calculation of a ward specific probability of death. Once patients were discharged from hospital the per day probability of death also included relative probability of death day based on discharge location of the patient: community hospital, residential or nursing home or home (see Table S1).

**Methods S2: Wards movements and discharge destination**

SINAP include details of the ward that stroke patients are first admitted to and subsequent movements in the first 72 hours, with details collected at each 24 hour follow-up for the following ward categories:

- Stroke unit (including HASU, CSC, PSC and DSC, but not specified)
- Medical assessment unit
- General ward
- Intensive care or critical care unit
- Other

These data were then used to calculate the percentage of patients admitted to each ward and daily transition probabilities between wards. The transition probabilities 48 hours to 72 hours were applied for day three to day 90 if patients were still in hospital as they were considered the most likely to be representative of transition probabilities for 72 hours to discharge.

There was no equivalent to SINAP before the reconfigurations took place for any of the regions (SINAP has data from April 2010). The only available data are from the National Sentinel Stroke clinical Audit that ran every two years. It contains the specific ward of admission only for patients admitted to a stroke unit – all other patients have no details on what ward they were first admitted to. The percentage of patients admitted to a stroke unit was updated to reflect the percentage ‘before’. The transition probabilities for the ‘before’ period for all wards other than stroke unit and medical assessment unit remained the same as ‘after’ given that there is no better data available. For example, 2% of patients were admitted to general medical wards ‘after’, so it was assumed 2% of patients were also admitted to general medical wards ‘before’. The only change made to transition probabilities was the a reduction in the percentage of patients admitted to stroke wards and an equal increase in percentage of patients admitted to medical wards instead

Discharge destination was calculated from the percentage of patients discharged to four locations: publicly financed residential care or nursing home, private nursing home, home with community rehabilitation, and NHS community hospital (see Table S3). All other patients with other discharge codes were removed from this analysis only. It was assumed patients were discharged to a community hospital if their discharge destination was “Other NHS Hospital” but they had no subsequent admission for the next day as community hospitals do not submit data to HES. If the discharge destination was “Other NHS hospital” and a new admission (new episode in HES) occurred within a day we assumed that this was a transfer between hospitals.

Given that data for changes in Barthel index before and after reconfigurations were only available for South London from the SLSR we made the conservative assumption that the same improvements in functioning also occurred in GM and England. A probabilistic sensitivity analysis was run to test the impact of changing the assumption so that the same improvements in functioning that were seen in London also occurred in GM but did not take place in the rest of England. Instead the Barthel index breakdown for the rest of England remained the same after the reconfiguration as it was before the reconfiguration.

There is limited evidence regarding the probability of patients moving into a care home following stroke. The transition probabilities for moving into a care home in the 10 year model were taken from the SLSR, so reflect the probability of moving into a care home in South London only. Other nine month probabilities of care home admission over 90 days quoted in the literature for older patients from throughout England range from 0.9%^22^ to 0.26%^24^ although these are not specifically for stroke patients. The impact of using alternative values has been tested in a deterministic sensitivity analysis.

The cost of transfers for patients between hospitals was originally included in the London HASU tariff so this has not been included as an additional cost. There is the possibility that transfers did result in an additional cost to the health service. The additional cost per transfer was included as a deterministic sensitivity analysis at £43 per transfer^25^. The percentage of patients that transfer between hospitals was calculated from HES.

More detailed evidence from previous analyses found that in London admission to ITU was 4% before the reconfigurations compared to 2% after^7^. There is no information in the Sentinel audit on ward admission so this comparison could not be made in the current analysis. A deterministic analysis was conducted to determine what impact increasing the percentage of people admitted to ITUs before the reconfiguration in London and Manchester to 4% would have on the results.

Increasing the cost of a day on the HASU by 50% was also included as a deterministic sensitivity analysis.

Table S6: Results of deterministic sensitivity analysis London per 1000 patients

|  | London | | | England | | | DID |
| --- | --- | --- | --- | --- | --- | --- | --- |
|  | Before | After | Difference | Before | After | Difference |  |
| Movements to Nursing home per 90 days = 0.9% probability. 10 year results | | | | | | | |
| QALYs | 2832 | 3352 | 520 | 2709 | 3177 | 468 | 52 |
| Costs | £44 506 798 | £43 526 204 | -£980 593 | £44 588 151 | 42 514 328 | -£2 073 823 | £1 093 230 |
| NMB £30 000 per QALY |  |  | £16 567 |  |  | £16 102 | £465 |
| Movements to Nursing home per 90 days = 0.26% probability. 10 year results | | | | | | | |
| QALYs | 3120 | 3698 | 579 | 2983 | 3505 | 522 | 57 |
| Costs | £34 160 157 | £32 565 792 | -£1 594 365 | £34 679 656 | £32 103 072 | -£2 576 584 | £982 219 |
| NMB £30 000 per QALY |  |  | £18 962 |  |  | £18 227 | £735 |
| Additional cost for transfer between hospitals in London | | | | | | | |
| 90 day costs | £5 709 351 | £5 964 428 | £255 077 | £5 500 302 | £4 973 647 | -£526 655 | £770 036 |
| QALYs – 10 years | 2928 | 3468 | 541 | 2800 | 3285 | 485 | 55 |
| Costs – 10 years | £39 604 757 | £38 333 585 | -£1 271 172 | £39 943 000 | £37 660 758 | -£2 282 215 | £1 011 043 |
| NMB £30 000 per QALY |  |  | £17 489 |  |  | £16 839 | £650 |
| London – 4% ITU admissions before | | | | | | | |
| 90 day costs | £5 835 220 | £5 949 507 | £114 288 | £5 494 071 | £4 966 449 | -£527 622 | £641 910 |
| QALYs – 10 years | 2928 | 3468 | 541 | 2800 | 3285 | 485 | 55 |
| Costs – 10 years | £39 747 568 | £38 316 675 | £1 430 892 | £39 935 961 | £37 652 684 | -£2 283 277 | £852 385 |
| NMB £30 000 per QALY |  |  | £17 650 |  |  | £16 840 | £810 |
| HASU costs 50% more per day | | | | | | | |
| 90 day costs | £5 704 557 | £6 562 152 | £857 595 | £5 494 071 | £4 966 449 | -£527 622 | £1 997 861 |
| QALYs – 10 years | 2928 | 3468 | 541 | 2800 | 3285 | 485 | 55 |
| Costs – 10 years | £39 599 318 | £39 010 989 | -£588 328 | £39 935 961 | £37 652 684 | -£2 283 277 | £1 694 949 |
| NMB £30 000 per QALY |  |  | £16 806 |  |  | £16 840 | -£34 |

LOS=Length of stay; QALYs = Quality Adjusted Life Years; DID= Difference-in-difference; NMB = Net Monetary Benefit

Table S7: Results of deterministic sensitivity analysis Greater Manchester (GM) per 1000 patients

|  | Greater Manchester | | | England | | | DID |
| --- | --- | --- | --- | --- | --- | --- | --- |
|  | Before | After | Difference | Before | After | Difference |  |
| Movements to Nursing home per 90 days = 0.9% probability. 10 year results | | | | | | | |
| QALYs | 2662 | 3177 | 515 | 2658 | 3157 | 499 | 16 |
| Costs | £44 115 237 | £42 120 777 | -£1 994 459 | £43 784 539 | £42 204 647 | -£1 579 892 | -£414 567 |
| NMB £30 000 per QALY |  |  | £17 448 |  |  | £16 542 | £907 |
| Movements to Nursing home per 90 days = 0.26% probability. 10 year results | | | | | | | |
| QALYs | 3120 | 3698 | 579 | 2927 | 3483 | 556 | 18 |
| Costs | £34 354 718 | £31 722 101 | -£2 632 617 | £34 062 280 | £31 858 690 | -£2 203 589 | -£429 028 |
| NMB £30 000 per QALY (per patient) |  |  | £19 847 |  |  | £18 881 | £966 |
| Manchester – 4% ITU admissions before | | | | | | | |
| 90 day costs | £5 724 473 | £5 213 429 | -£511 044 | £5 473 849 | £4 905 472 | -£532 376 | £21 333 |
| QALYs – 10 years | 2750 | 3286 | 536 | 2747 | 3264 | 517 | 18 |
| Costs – 10 years | £39 685 105 | £37 234 169 | -£2 450 936 | £39 220 696 | £37 374 815 | -£ 1 845 881 | -£605 055 |
| NMB £30 000 per QALY |  |  | £18 521 |  |  | £17 367 | £1 154 |

LOS=Length of stay; QALYs = Quality Adjusted Life Years; DID= Difference-in-difference; NMB = Net Monetary Benefit

Figure S2: Cost-effectiveness acceptability curve for if improvements in functioning are seen in London and Manchester but not in the rest of England after the reconfigurations
